# Effect of COVID-19 response policies on walking behavior in US cities

**DOI:** 10.1038/s41467-021-23937-9

**Published:** 2021-06-16

**Authors:** Ruth F. Hunter, Leandro Garcia, Thiago Herick de Sa, Belen Zapata-Diomedi, Christopher Millett, James Woodcock, Alex ’Sandy’ Pentland, Esteban Moro

**Affiliations:** 1 Centre for Public Health, Queen’s University Belfast, Belfast, UK; 2 Center for Epidemiological Research in Nutrition and Health, Universtiy of São Paulo, São Paulo, Brazil; 3 Healthy Liveable Cities Group, Centre for Urban Research, RMIT University, Melbourne, VIC Australia; 4 Public Health Policy Evaluation Unit, School of Public Health, Imperial College London, London, UK; 5 Centre for Diet and Activity Research, MRC Epidemiology Unit, University of Cambridge, Cambridge, UK; 6 Connection Science, Institute for Data Science and Society, MIT, Cambridge, MA USA; 7 Department of Mathematics and GISC, Universidad Carlos III de Madrid, Leganés, Spain

**Keywords:** Risk factors, Applied mathematics

## Abstract

The COVID-19 pandemic is causing mass disruption to our daily lives. We integrate mobility data from mobile devices and area-level data to study the walking patterns of 1.62 million anonymous users in 10 metropolitan areas in the United States. The data covers the period from mid-February 2020 (pre-lockdown) to late June 2020 (easing of lockdown restrictions). We detect when users were walking, distance walked and time of the walk, and classify each walk as recreational or utilitarian. Our results reveal dramatic declines in walking, particularly utilitarian walking, while recreational walking has recovered and even surpassed pre-pandemic levels. Our findings also demonstrate important social patterns, widening existing inequalities in walking behavior. COVID-19 response measures have a larger impact on walking behavior for those from low-income areas and high use of public transportation. Provision of equal opportunities to support walking is key to opening up our society and economy.

## Abstract (web summary)

Mobility restrictions implemented to reduce the spread of COVID-19 have significantly impacted walking behavior. In this study, the authors integrated mobility data from mobile devices and area-level data to study the walking patterns of 1.62 million anonymous users in 10 US metropolitan areas.

## Introduction

In response to the COVID-19 outbreak, many countries have implemented interventions to induce mobility restrictions and force their citizens to stay at home (i.e., confinement) to reduce the transmission rate and prevent health services from being overwhelmed. As a result, at timepoints during the pandemic, over half of the world’s population have been committed to stay at home for different periods of time, causing major disruptions to their daily lives. As time has elapsed and countries are learning how to live with COVID-19, countries ebb and flow out of confinement and other social distancing policies, trying to maintain a difficult balance between reducing the transmission rate of the virus and preserving a functioning economy. Although the key to containing the spread of the virus, such restrictions have caused large-scale disruption to our normal lives and active living. Preliminary descriptive studies have shown a large decrease in country-level physical activity because of the stay-at-home recommendations and strict lockdown measures^1^. Even before the COVID-19 pandemic, physical inactivity was highly prevalent—27.5% of adults and 80% of adolescents worldwide did not meet the recommended levels for health benefits^2,3^—and the fourth risk factor contributing 6% to global mortality (WHO, 2014). If we assume at least half of the world’s population has some level of lockdown restrictions in place due to the COVID-19 pandemic, then physical inactivity rates could reach more than 1.1 billion people^4,5^.

Physical activity has a range of benefits that includes physical and mental health and well-being, strengthening of social interactions and social capital, and economic and environmental returns^6,7^. Walking is the most popular and accessible type of physical activity behavior. For instance, in the US, walking is consistently the most prevalent leisure-time physical activity and the most frequently reported physical activity among adults who meet public health physical activity recommendations^8^. In the US, 50.0% [95% CI 49.1–51.0%] of adults engage in leisure walking activity and around 29.4% [95% CI 28.6–30.3%] in utilitarian (transportation, shopping, routine) walking^8^. Despite these figures, only half of Americans self-report that they meet a minimum of 30 min of walking five or more times per week^9^. Utilitarian walking is in general 20% shorter but more prevalent than leisure walking^10^.

There is no doubt that mobility restrictions implemented to reduce the spread of COVID-19 have impacted walking behavior, but the magnitude and spatio-temporal aspects of these changes have not been thoroughly investigated yet. Much less is known on the differential impacts of COVID-19 response measures on the walking behavior of population subgroups. The COVID-19 pandemic is occurring in a context of social and economic inequalities in existing noncommunicable diseases and the social determinants of health^11^. The impact of COVID-19 on health inequalities will be driven by both virus-related infection and mortality rates and the consequences of the policy responses undertaken. For instance, different population subgroups are likely to have distinct experiences of lockdown according to their access to green space and urbanity, with unequal effects on their physical and mental health^11^. Given the large pre-pandemic inequalities in drivers and patterns of walking behavior, it should be expected that the impact of COVID-19 response measures on walking would show large variability between socio-demographic groups. Walking activity is unequally distributed among different socio-demographic groups. Men are more likely to walk for utilitarian purposes, but less likely for leisure, than women^8^. The prevalence of utilitarian walking decreases with increasing age, whereas the prevalence of leisure walking peaks among older adults. Prevalence of walking in either context tends to increase with increasing education level and with decreasing adiposity^12^. At the same time, COVID-19 pandemic and response measures had different effects across racial and economic groups^13^. Higher COVID-19 death rates were found in the most disadvantaged areas (proportion of persons living in poverty, proportion of crowded households, and concentration of extreme racial and socioeconomic segregation) and areas with the largest populations of people of color (proportion of the population that is non-white, non-Hispanic).

Understanding how COVID-19 response measures have affected the walking behavior of populations and its distinct subgroups is important information to help devise strategies to prevent the potential health and societal impacts of declining walking levels^1^. To do so, a deep, at-scale understanding that goes beyond mere descriptive analysis is required. Indirect impacts of the pandemic on health, including through reductions in physical activity^14^, have been described, but mainly based on small-scale, self-report surveys and qualitative methods (see refs. ^15–17^), which have well-documented biases^18^ and, sometimes, limited generalizability. These studies have also tended to focus on overall physical activity levels, with little attention to date on specific activities such as walking. In a recent systematic review^19^, of the included 45 studies that explored changes in physical activity behavior in healthy adults, only four used device-based measures, and the sample sizes for the four studies ranged from 18 to 2289 adults^20–23^. Furthermore, the lack of specificity in distinguishing between utilitarian and recreational walking weakens the power of previous walking behavior analyses, with subsequent implications on policy recommendations.

In this study, we make a unique contribution to the current literature by analyzing changes in walking behavior using mobile phone’s geolocalized mobility data from more than 1.6 million people in urban areas, which provides a step-change in our understanding of walking behavior during the pandemic to date. We have defined walking behavior as speeds up to 2 m/s (see “Methods” section). Our intention is not to capture all possible walks but to use a proxy measure to detect an important population-level change in walking behavior during the pandemic period. Mobile phones are a powerful tool with which to study large-scale population dynamics, revealing patterns of human movement at greater temporal and spatial granularity, while ensuring anonymity and user privacy. Smartphones with in-built accelerometers enable 24-h automatic recording of physical activity, providing a scalable tool to measure and quantify walking behavior^24^. We integrate anonymized and privacy-enhanced data from mobile devices and area-level data to study the walking patterns of 1.62 million anonymous users in 10 metropolitan areas in the US to investigate the effect on walking of COVID-19 response measures. The data covers the period from mid-February 2020 (pre-lockdown) to late June 2020 (easing of lockdown restrictions) in the following metropolitan areas in the US: New York, Los Angeles, Chicago, Boston, Miami, Dallas, San Francisco, Seattle, Philadelphia, and Washington DC. Using simple algorithms (see “Methods”), we detected when users were walking, measured the distance walked and time of the walk, and classified each walk as recreational or utilitarian. We also investigated walking levels during different periods of the COVID-19 response measures by population subgroups according to demographic, social, health, and built environment aspects of the places where they live.

## Results

### Effect of measures on utilitarian and leisure walking

**Fig. 1:**
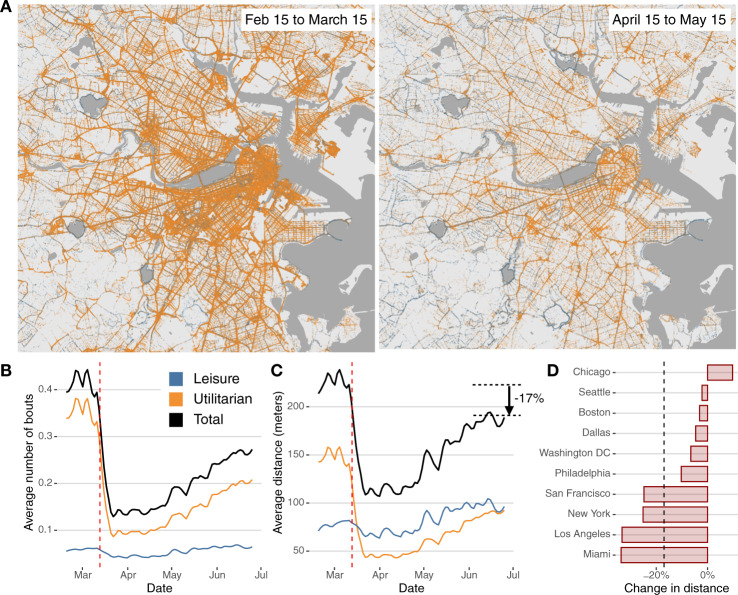
Decrease in walking behavior during the pandemic. **A** Geolocations of leisure and utilitarian walks in the Boston area before (left) and after (right) the introduction of COVID-19 response measures. **B** Total (black) daily average number of bouts of walking by day and user in the 10 metropolitan areas, compared with those for utilitarian and leisure walks. Vertical (red) dashed line indicates March 13, 2020, the declaration of a national emergency. **C** Same as in (**B**) but for total distance walked. **D** Relative change in distance walked by the city between the pre-lockdown (Feb 15, 2020 to March 15, 2020) and post-lockdown (June 2020). Maps were produced in *R* using the TIGER shapefiles from the U.S. Census Bureau^42^.

Although COVID-19 response measures differed between metropolitan areas, in most of the US, social distancing started after the declaration of a national emergency on March 13, 2020 and the schools and nonessential business closings during the following week. As we can see in Fig. 1, that translated into a substantial decrease of ~70% in the number of walks (bouts) and 50% in the average distance walked in all metropolitan areas. Since mid-April 2020, walking steadily increased although even after resuming some commercial and business activities, walking was still around 18% below the pre-pandemic level. Walking decreased considerably in Miami (−33.7%), Los Angeles (−33.3%), New York (−25.1%), and San Francisco (−24.8%), whereas in Chicago (+9.7%), Seattle (−2.2%), and Boston (−3.1%) the distance walked was even slightly higher or similar than before the pandemic. This could be also the reflection of much better weather conditions, particularly in places like Chicago, Seattle, or Boston.

To understand the reasons for the substantial impact of the COVID-19 response measures in walking behavior, we investigated the origin and nature of walking behavior in our cities. Our analysis showed that 70% of users undertook at least one bout of walking per week before COVID-19 response measures were introduced (March 15, 2020) and 54% walked at least 10 min per week. Our findings are comparable to the National Health Interview Survey (NHIS) 2010 results (self-reported 61%^8^). On average, individuals did 0.52 bout of walking per day, similar to what was observed in the NHIS 2017 (0.49%^25^). We found that only 3.1% of users walked at least 30 min in 5 or more days per week (which were the national physical activity recommendations at that time), comparable with 6% reported in the NHIS^12^. Walks were typically short, with an average distance of 701.79 m [95% CI 701.59, 702.0] and a duration of 11.4 min [95% CI 11.39, 11.41].

**Fig. 2:**
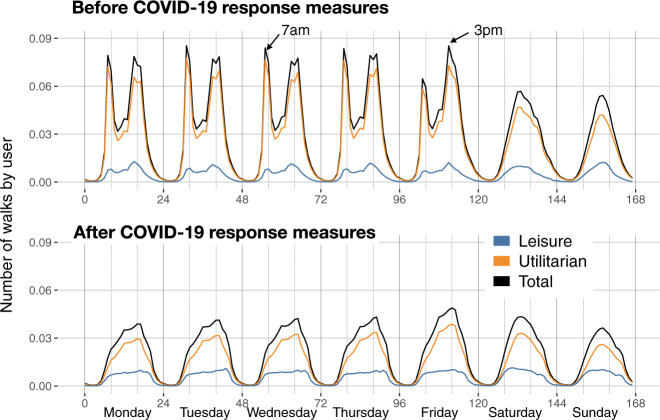
Temporal patterns of walking behavior. Panels show the average number of walks by user for each hour and day of the week, and those for leisure and utilitarian purposes. The upper panel corresponds to the temporal pattern before COVID-19 response measures and the lower panel after COVID-19 response measures.

To understand the nature of the walks, we used a simple classification of the bouts in utilitarian and leisure walking based on the distance walked and their final destination (see “Methods”). We found that most of the walks (75.45%) were utilitarian, but leisure walks were longer (1495.24 m, 95% CI 1494.74, 1495.75) than utilitarian walks (432.78;m, 95% CI 432.66, 432.91). Thus, a larger proportion of the distance walked in US metropolitan areas corresponded to leisure walking. During weekdays, most of the utilitarian walks occurred around 7 am and from 3 pm to 5 pm, while they concentrated mostly around lunch time during the weekends (see Fig. 2).

As we can see in Fig. 1, the impact of COVID-19 response measures significantly impacted utilitarian walking (−72.3% in the distance traveled directly after the declaration of a national emergency). Figure 2 shows a sharp decrease in the number of walks around 7 am and 3 pm, and most of the walking activity during weekdays resembled that of weekends. Our results suggest that the large impact of COVID-19 response measures in walking was mostly due to the interruption of our working, shopping, and dining activities. Even after the easing of the restrictions (after mid-May 2020), utilitarian walking was still −39.2% (in distance traveled) less than before the pandemic. Leisure walking was not affected as much (Fig. 1C) and recovered and surpassed the levels before the pandemic.

### Impact by socio-demographic groups

**Fig. 3:**
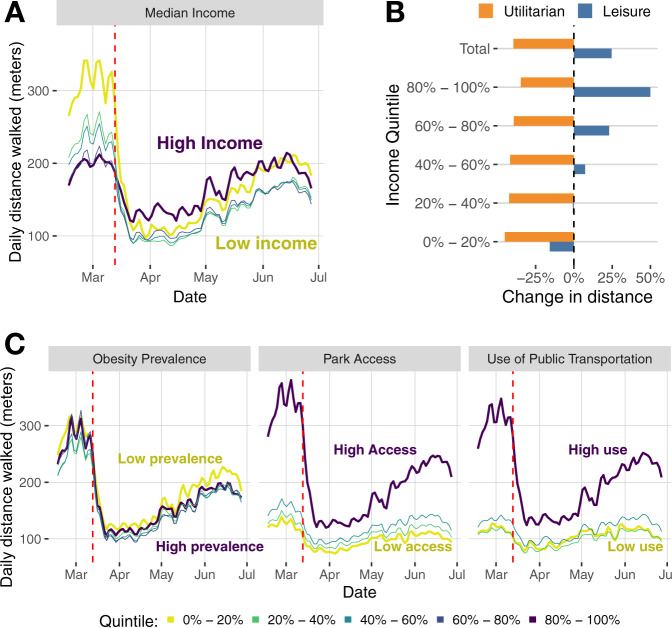
Change in walking by different socio-demographic groups. **A** Average daily distance walked by different area-level income categories (quintiles). **B** Change in utilitarian or leisure walking for the same area-level income categories, including the total. **C** Evolution of the average distance walked by day by different categories (quintiles) for area-level obesity prevalence, park access, and use of public transportation.

Due to the anonymous nature of our location data, we examined the socio-demographic differences at the area level, which are defined by census tracts where users live. Figure 3 shows the changes in the total number of walks and distance walked across census tracts with different socio-demographic features. Before the introduction of COVID-19 response measures, we can clearly see that areas of low-income, low-obesity prevalence, high park access, or high use of public transportation had higher walking activity. After the introduction of COVID-19 response measures, this was still true for obesity prevalence, park access, or use of public transportation, although the relative change differed across these groups (see Fig. 3C). A similar small relative change between census tracts is also found for other demographic dimensions like fraction of old (>64-year-old people) or Black people living in the area (see Supplementary Fig. 9).

People living in areas with high use of public transportation experienced a sharp decrease in walking activity compared to those living in areas of low use, probably due to the reduction of walking to transportation. Different temporal patterns can be according to area-level income (see Fig. 3A): before COVID-19 response measures, people living in high-income areas walked less than those living in low-income areas; however, the pattern reversed after COVID-19 response measures. Furthermore, walking levels recovered to pre-COVID-19 measures in high-income areas, whereas low-income areas were still well below pre-COVID-19 levels. Figure 3B provides insights into the potential reasons for the differential observed across places with different income levels. Utilitarian walking has decreased across all income quintiles between February 2020 and June 2020. However, people living in high-income areas have increased their leisure walking considerably (49% for the highest income quintile). This result might be related to the fact that high-income individuals had more opportunities for social distancing by staying and working at home, and also more free time and opportunities to engage in leisure walking. The substitution of utilitarian for leisure walking was not present in low-income groups and, as a result, the COVID-19 response measures have had a strong impact on their walking behavior.

Using multivariate linear mixed regression, we investigated the combined effect of area-level socio-demographic (income, race, age), health (obesity prevalence), and environmental (park access and use of public transportation) variables on the amount of walking pre-pandemic and after COVID-19 response measures were introduced. In our models, we have also included a fixed factor by the city to account for the different nature of walking across cities (e.g., weather, infrastructure, etc.) and the different impact of interventions during the first wave. Table 1 shows the use of public transportation was the most relevant to understanding walking activity: one standard deviation in the use of public transportation increased the amount of individual walking by 54 m per day. Areas with higher income, more black people, higher obesity prevalence, or with a larger fraction of 64-year-old or older walked less on average, while those with higher access to park walked more. After the introduction of COVID-19 response measures, the use of public transportation was still the most impactful variable to understand the walking activity. But, as noted before, the effect of the variables was different from the situation before COVID-19 response measures were introduced: area-level income is no longer a significant variable, and areas with more park access seemed to walk more because they engaged more in leisure walking. However, areas with more black people or higher obesity prevalence walked less on average.

**Table 1:** Regression results for the multivariate linear mixed model (see Eq. (1) in “Methods”) to explain the average distance walked (in meters) by individuals in different areas as a function of the demographic properties of that area.

	Average distance walked	
	Before COVID response	After COVID response
Median income	−29.205^***^ (1.627)	−1.586 (1.179)
Fraction of black people	2.745^**^ (1.396)	−2.758^***^ (1.012)
Fraction of users of public transportation	54.170^***^ (1.502)	33.070^***^ (1.089)
Fraction of people older than 64 years	−0.331 (1.307)	2.088^**^ (0.948)
Park access	15.691^***^ (1.350)	8.058^***^ (0.978)
Obesity prevalence	−20.703^***^ (2.343)	−11.889^***^ (1.699)
Constant	315.572^***^ (5.640)	239.919^***^ (4.088)
City fixed effect	Yes	Yes
Observations	9111	9111
*R*^2^	0.764	0.699
Adjusted *R*^2^	0.763	0.699
Residual std. error (df = 9095)	100.212	72.641
*F* statistic (df = 15; 9095)	1960.365^***^	1408.228^***^

*Note*: ^*^p < 0.1; ^**^p < 0.05; ^***^p < 0.01.

The table shows the standardized coefficients for the regression models before and after the COVID-19 response measures were introduced. See “Methods”.

## Discussion

COVID-19 response measures resulted in large-scale disruption to our daily walking behavior. Our findings reveal that in all ten metropolitan areas investigated, utilitarian walking decreased dramatically at the beginning of the lockdown restrictions owing to reductions in the needs and opportunities to walk to work, to public transport, to shop, and to other amenities. The decrease in walking for leisure was less pronounced in general and, in some areas, it has surpassed levels before the pandemic.

### The tale of two pandemics

Our findings also demonstrate an important differential impact on walking behavior according to area-level demographic, social, health, and built environment factors, exacerbating existing inequalities. We show that COVID-19 response measures had a larger impact on walking behavior for those from socially disadvantaged areas, with larger fractions of public transportation users, worse obesity indicators, and a physical environment that is less conducive to walking. On the contrary, high-income areas have substituted utilitarian walking activity for more leisure walking, sometimes reaching even higher levels of walking than before the pandemic.

The COVID-19 pandemic has caused disruption at scale and requires an unprecedented level of response if we are to combat the emergent findings showing the impact on walking behavior and the widening of inherent inequalities. The argument of a physical inactivity pandemic has been made before^4^, and some have described the current COVID-physical inactivity interaction as a tale of two pandemics^26^. But COVID-19 has shown us what a political response to a real pandemic looks like. We have witnessed determined and swift actions of governments around the world to the public health threat from COVID-19. And there is an opportunity for the same governmental determination to take radical decisions on walking behavior and tackling inherent inequalities. For example, COVID-19 response measures in Milan, Paris, and London, included pedestrianizing streets and expanding cycle lanes, facilitating COVID-19-safe transport during the crisis, while enhancing economic activity and quality of life afterward^27^.

The provision of equal opportunities to support walking could be key to opening up our society and the economy. For example, our findings showed significant changes to utilitarian walking behavior, and with social distancing restrictions placed on local public transport, provision of environments to support utilitarian walking (and indeed cycling) provides a means of opening up the economy through the return to work, and a move from home working to the office environment to support city center economy. In the US, public transport use is typically higher in low-income communities^28^, which according to our findings suffered the largest decrease in walking behavior. Care must be taken to ensure that provision of public transport is safe, including COVID-19 mitigation strategies such as limiting the number of users, ensuring physical distancing, mandatory use of face coverings by passengers, provision of hand sanitizer, and regular cleaning of vehicles touch-points, seating etc. Albeit, the implementation of such strategies will have profound economic impacts for public transport providers.

Provision of support for leisure walking facilitates good health and well-being during social distancing and lockdown restrictions, and long-term, reduces the risk of obesity and noncommunicable diseases, which are significant risk factors for COVID-19^14^. Supportive environments include, for example, the availability of infrastructure such as access to green space (e.g., keeping public parks open) to support walking and other physical activity opportunities.

Our findings have provided evidence of the impact of COVID-19 responses on short-term inequalities for walking, and walking opportunities. If significant action is not taken, then these inequalities will likely widen in the longer term. For instance, although those residing in areas with the highest park access scores suffered the largest reductions in walking behavior, their average daily walking distance started to recover sooner, while distance walked by those living in areas with less access to parks did not recover at the same pace. It is important that the public health community consider what adaptive and mitigation measures can be introduced to limit the effect and support recovery.

### Opportunities for tactical urbanism

Some cities, especially in Europe, have sought to mitigate the impact of COVID-19 in walking behavior through temporary measures, including extending footpaths, protecting commuters using public transit through social distancing measures, pop-up cycle lanes. The impact of these measures on walking behavior, especially on their disparities, warrants careful evaluation. Governments in many settings have indicated that these changes will be permanent and probably will influence walking behavior and healthy urban planning for decades. This highlights the opportunity that the COVID-19 pandemic presents for restructuring societies to meet health and environmental goals, including through transport planning. With the emergence of candidate vaccines and substantial normalization of societies likely by the summer, there remains a crucial opportunity to build on this momentum to make permanent changes to our environments to encourage and support greater walking behavior.

Our study provides valuable methodological innovation and data to quantify walking patterns and inform future city planning and policy decisions. Mobile phone data can be used to inform local policies and practice in near to real time, disentangling implications of different aspects and phases of COVID-19 response strategies for different forms of walking behavior (i.e., utilitarian and recreational) to help tailor future policies and response measures that can provide suitable walking environments to support the health and economic futures of communities.

In Table 2, we outline a range of possible low-cost interventions and policies that could increase utilitarian and recreational walking behavior and tackle the inequalities highlighted above^29^. Some examples include creating pop-up footpaths and widening pavements which provide more opportunities to walk. Reducing speed limits in urban areas to promote pedestrian safety, and simple adaptations to adjust the timing of traffic lights to favor pedestrians provide low-cost solutions to support walking. In the long term, if made permanent, these interventions also provide an opportunity to improve population health and well-being. Other co-benefits may also include reduced air and noise pollution, reduced risk of traffic collisions and casualties, reduced risk of noncommunicable diseases, promoting social equity, and reducing the demand on our health services. We, therefore, have a unique opportunity to improve our urban environments to support and enable walking and physical activity, particularly for our most vulnerable communities.

**Table 2:** Examples of tactical urbanism strategies that can lead to permanent solutions to increase utilitarian and leisure walking behavior and reduce inequalities.

(1) Expanding pedestrian spaces and footpath networks
• Programs to encourage walking
• Widen and lengthen pavements and improve connections to promote traffic safety
• Reduce pressure on parks and public spaces by widening pavements in the surrounding area
• Facilitate access to vulnerable groups who would otherwise have to walk in worse environments (e.g., unsafe streets; crowded footpaths)
• Expand pedestrian infrastructure using existing proposals
• Expand pedestrian access to the provision of safe public transport
(2) Providing recreational paths for walking
• Create and expand existing networks
• Establish social networks to enable and support walking
(3) Adapting parks and public spaces
• Keep open large public spaces (e.g., parks)
• Ensure that parks and public space users have access to water, hygiene, and sanitation.
• Implement and enforce measures to increase perceived and objective safety against violence, harassment.
(4) Adapting traffic lights, signage, and speed limits
• Replace button-activated traffic lights with automatic systems.
• Adjust the timing of traffic lights to favor pedestrians
• Reduce speed limits throughout the city to promote pedestrian safety
• Implement and enforce measures to increase pedestrian safety on traffic

Adapted from refs. ^27^ and ^29^.

### Strengths and limitations

We investigated the impact of the COVID-19 pandemic on walking behavior in ten of the largest US metropolitan areas using high-resolution mobility data. These data encompass more than 1.6 million people and a wide variation in socio-demographic, health, and built environment aspects. Our paper investigates changes in walking behavior in response to COVID-19 restriction measures from multiple societal angles. The results align with a priori expectations, but the magnitude of the disparities was previously unknown. Our findings confirm our expectations and reveal the magnitude of the differences, giving a more nuanced picture of how socioeconomic disparities can affect population-wide recovery from COVID-19. In particular, our finding regarding utilitarian walking is unique and important because: (a) this is the first paper to investigate the impact of the pandemic on leisure and utilitarian walking behavior separately, and (b) the finding regarding utilitarian walking behavior has important policy implications as cities struggle to find a balance between COVID-19 restriction measures to control the spread of the virus and keeping the economy open. Safe access to, and use of, public transport are urgently required. The recent systematic review by Stockwell et al.^19^ also emphasizes the novelty of our findings in the current pandemic context, arguments that we have rehearsed earlier in this paper. The US is an important exemplar internationally due to having the highest number of COVID-19 cases globally, its variations across metropolitan areas in existing active travel infrastructure and pre-pandemic walking behavior, and stark inequalities in walking behavior and health outcomes that should be explicitly considered within the COVID-19 response.

Walking is the most common physical activity, but our dataset may fail to capture time spent walking when users did not carry their phones. As mentioned earlier, we have purposefully utilized a pragmatic definition of walking behavior to detect the change in leisure and utilitarian walking, making optimal use of a very large population dataset captured during the pandemic period, enabling us to address questions that are not possible to answer using self-reported data or data from wearable devices. Further, data from wearable devices would have introduced well-known systemic biases, largely excluding the very low-income groups and inequalities that we set out to address in this study. We acknowledge that and systematic differences may exist in wear time in our dataset based on individual factors such as gender and age. Further, if we had demographic data associated with individuals’ mobility data, we could directly examine walking behavior across demographics at the individual level. However, the anonymous location data does not contain demographic information. Our walking speed of 2 m/s is derived from the literature and we have included a sensitivity analysis to investigate the impact of different walking speeds on our findings. However, we acknowledge that a certain small fraction of the detected “walking behavior” could be other activities, for example, slow-moving cars in city centers.

It is evident that the COVID-19 pandemic has inextricably changed walking behavior in US cities. Such large-scale alterations in walking patterns were inconceivable pre-COVID-19 pandemic. However, most importantly, our findings show that inequalities in walking opportunities among communities and neighborhoods have been reinforced, and new inequalities due to the interaction with COVID-19 response measures emerged. The experience of COVID-19 is neither shared equally across our communities and neighborhoods nor is its impact. As Marmot and Allen^30^ stated COVID-19 exposes the fault lines in society and amplifies inequalities. Our findings highlight walking inequality as an important indicator of disparities in the population and identify walking poor areas, whose residents importantly are most at risk from COVID-19. The residents of these areas are set to benefit most from interventions and policies to promote walking and other physical activities. The methods applied in this work and our findings can help us to understand the prevalence, spread, and effects of walking within and across cities, countries, and subgroups and to design communities, policies, and actions that promote greater walking in a COVID-19 secure world.

## Methods

### Mobility data

The privacy-enhanced mobility data were obtained from Cuebiq, a location intelligence, and measurement company. The dataset consists of anonymized records of GPS locations from anonymous users that opted-in to share the data anonymously in ten metropolitan areas over a period of 5 months, from February to June 2020. Data were shared under a strict contract with Cuebiq through their Data for Good COVID-19 Collaborative program where they provide access to de-identified and privacy-enhanced mobility data for academic research and humanitarian initiatives only. All researchers were contractually obligated to not share data further or to attempt to re-identify data. Mobility data are derived from anonymous users who opted to share their data anonymously through a General Data Protection Regulation (GDPR) and California Consumer Privacy Act (CCPA) compliant framework. Additionally, we obtained IRB exemption to use the mobility data from the MIT IRB office through protocols #1812635835 and its extension #E-2962.

Our sample dataset achieves broad geographic representation. Although the population and number of anonymous devices detected in the real data by census tract area is highly correlated (Pearson’s correlation of 0.66), post-stratification techniques were implemented to ensure the representativeness of the data at the level of population^31^. See Supplementary Note 1 for more details about the post-stratification method used and further details about our panel or users.

### Other data

Demographic data (median income, fraction of black people, fraction of users of public transportation, and fraction of people older than 64 years of age) by census tract was obtained through the census ACS 5-year 2013–2018 datasets^32^. Obesity prevalence by census tract is given by the Center for Disease Control 500 cities estimations^33^. Park access was obtained from the City Health Dashboard^34^.

### Multivariate linear mixed model

To study the combined effect of the demographic, built environment, and health variables on walking behavior, we used multivariate mixed linear regression to explain ${\overline{d}}_{\alpha ,i}$, the average distance walked by people living in a census tract *α* in city *i* as a function of those variables

1 $$\begin{array}{lll}{\overline{d}}_{\alpha ,i}& \sim &{{\rm{income}}}_{\alpha ,i}+{{\rm{black}}}_{\alpha ,i}+{\rm{public}}\,{{\rm{transportation}}}_{\alpha ,i}\\ &&+{\rm{older}}\,6{4}_{\alpha ,i}+{{\rm{park}}}_{\alpha ,i}+{{\rm{obesity}}}_{\alpha ,i}+{{\rm{MSA}}}_{i}+{\varepsilon }_{\alpha ,i}\end{array}$$

where *ε*_*α*,*i*_ is regression error and MSA_*i*_ is a fixed factor by Metropolitan Statistical Area (MSA) to account for the different nature of walking across cities (e.g., weather, infrastructure, etc.) and the different impact of interventions during the first wave. Results are presented in Table 1, where ${\overline{d}}_{\alpha }$ is calculated before and after COVID response measures (March 15). We have also tested the potential bias of the spatial autocorrelation of our variables in our results. As we can see in Supplementary Note 4 our results are not considerably affected by the spatial structure of the variables.

### Walk detection

To detect walk activity from the mobility data, we used a methodology based on speed threshold and map-matching techniques^35,36^. Walk bouts were extracted from consecutive locations in which the speed between them did not exceed typical walking speeds (2 m/s)^36–38^. Due to the geospatial accuracy of the data, we discarded walks of distance less than 50 m.

To minimize the impact of trajectories that could be misinterpreted as walking behavior, we only considered trajectories with geolocations that happened close to potential pedestrian areas. Using Open Street Maps we define those areas as the ones up to 20 m from a secondary or tertiary road, residential or living streets, pedestrian, footway, track, or path^39^. This definition excludes highways, motorways, or trunks, where slow traffic or congestion could be mistaken as walks. We also considered those geolocations that happened within parks. Note that this strict definition of areas in which walks are possible to exclude some of them where walking activity could be happening, like in parking areas, buildings, or other large indoor spaces.

We have also tested extensively the sensitivity of our results towards our walk detection parameters (speed of 2 m/s) and the possibility that the walks could be the slow parts of other mobility modalities (e.g., traffic jams, stops, or intersections in cities). As we can see in Supplementary Note 2, our results are robust and are not affected by these sensitivity tests. Finally, we have compared our results by city with other mobility reports (Apple Mobility)^40^, see Supplementary Note 3. Despite the different origins of the walking activity data, they overlap quite accurately in most areas, showing that our walk detection method reflects real walking activity in metropolitan areas.

### Walk classification

Walks have different purposes. The most common are recreational (leisure), transportation, shopping, or going to work. Although in some cases it might be difficult to separate them (a recreational walk may involve some shopping), most of the literature separates walks by distance and/or destination. Leisure and utilitarian walks have very different lengths^8^ and, of course, most of the leisure walks happen in outdoor spaces or close to walkers’ residences.

In our data (see Supplementary Fig. 1) during weekdays, most of the walks occurred around 7 am and from 3 pm to 5 pm. These walks were very short in length (below 750 m) and are probably related to rush hour (i.e., travels to transportation)^8^, which is known to make up a significant proportion of walking behavior in US cities. Further evidence is provided by the disappearance of those clusters of walks during the weekends, being replaced by a cluster of very short walks around lunch time (noon). Also, after COVID-19 response measures were introduced, these patterns disappeared and most of the walks happened from 4 pm to 6 pm and with distances further than 750 m. These findings suggest that there is a difference between walks below 750 m which likely correspond mostly to utilitarian walking.

On the other hand, walks have very different destinations. Even a walk longer than 750 m could end up in shopping, transportation, or work venue. Using a large database of 1.5 million points of interest (POI) from Foursquare we investigate the destination of the walks using the closest (up to 25 m) POI to the last point of the walk before COVID-19 response measures. Supplementary Table 1 shows the properties of the walks by different types of destinations. In all the cities, around 62% of the walks do not end up in a particular POI of our dataset, while for the rest the most frequent destination is food (7.93%), service (6.42%), transportation (5.19%), or outdoors (3.1%) categories. Shorter walks are to school (462.91 m), health (488.16 m), shopping (489.53 m), or service (496.10 m), while those without a destination (565.14 m), coffee/tea (529.94 m), work (523.86 m), or city/outdoors (522.27 m) destinations are longer. Our results are similar to those in ref. ^41^, where it was found that workplaces, transportation, food, groceries, shopping/services were the most frequent destinations. Because of this variability for those walks longer than 750 m, we classified them as leisure if they do not end up on a particular POI of our dataset or if they end up in City/Outdoors (which includes residential areas).

### Robustness of walk classification

Our results are robust regarding our criteria to define utilitarian walks. In particular, the large drop in the number of utilitarian walks after COVID-19 response measures were introduced and the fact that leisure walks have not decreased significantly is also observed for other distance thresholds (see Supplementary Fig. 2).

### Reporting summary

Further information on research design is available in the Nature Research Reporting Summary linked to this article.

## Supplementary information

Supplementary Information

Reporting Summary

## Data Availability

The data that support the findings of this study are available from Cuebiq through their COVID19 Data for good program, but restrictions apply to the availability of these data, which were used under licenses for the current study, and so they are not publicly available. Information about how to request access to the data and its conditions and limitations can be found at https://www.cuebiq.com/about/data-for-good/. Source anonymized aggregated data are publicly available on github: https://github.com/emoro/walking_COVID19.

## Code availability

Code to reproduce our results in the figures from the aggregated data is publicly available on github https://github.com/emoro/walking_COVID19.
