# Supplementary material for: Effect of COVID-19 response policies on walking behavior in US cities
Source: Nat Commun. 2021 Jun 16;12:3652. doi: 10.1038/s41467-021-23937-9 (PMC8209100; doi:10.1038/s41467-021-23937-9)
Supplement: Supplementary file 1 — Supplementary Information [file 41467_2021_23937_MOESM1_ESM.pdf]

# – Supplementary Information –
Effect of COVID-19 response policies on walking behavior in US cities

Ruth F. Hunter, Leandro Garcia, Thiago Herick de Sa,
Belen Zapata-Diomedi, Christopher Millet, James Woodcock,
Alex 'Sandy' Pentland, Esteban Moro

May 18, 2021

Supplementary Notes

Supplementary Note 1 Mobility data 2

Supplementary Note 2 Sensitivity analysis for detection of walks 2

Supplementary Note 3 Comparison with other datasets 3

Supplementary Note 4 Spatial regression models 3

Supplementary Note 5 List of software used 4

List of Supplementary Figures

1 Supplementary Figure 1 6
2 Supplementary Figure 2 7
3 Supplementary Figure 3 8
4 Supplementary Figure 4 9
5 Supplementary Figure 5 10
6 Supplementary Figure 5 11
7 Supplementary Figure 5 12
8 Supplementary Figure 6 13
9 Supplementary Figure 7 14

List of Supplementary Tables

1 Supplementary Table 1 7
2 Supplementary Table 2 15

Supplementary Note 1 Mobility data

The mobility data was obtained from Cuebiq, a location intelligence and measurement company. The dataset consists of anonymized records of GPS locations from users that opted-in to share the data anonymously in the ten of the largest metropolitan areas in the US over a period of 5 months, from February 2020 to June 2020. In addition to anonymizing the data, the data provider obfuscates home locations to the census block group level to preserve privacy. Data was shared in 2020 under a strict contract with Cuebiq through their Data for Good program where they provide access to de-identified and privacy-enhanced mobility data for academic research and humanitarian initiatives only. All researchers were contractually obligated to not share data further or to attempt to de-identify data. Mobility data is derived from users who opted in to share their data anonymously through a General Data Protection Regulation (GDPR) and California Consumer Privacy Act (CCPA) compliant framework.

For privacy reasons, our data is obfuscated around home to the level of Census Block Groups. Thus the attribution between the mobility data and home and workplaces happen at the level of Census Block groups. We estimate the home Census tract of the anonymous users as the one in which they are more likely located during nighttime (from 8pm to 4am). We have only included users for whom we were able to detect their homes before the lockdown. To minimize the effect of people leaving the city or users that disappear from the dataset, we have studied only the walking activity of a panel of users comprising of those for which we could detect their home Census Block group before the lockdown and that are still active in June 2020. For statistical and privacy reasons we only considered Census tracts which are home for more than ten anaonymous users.

Our sample dataset achieves broad geographic representation. Although the population and number of anonymous devices detected in the real data by census tract area is highly correlated (Pearson's correlation of 0.66), post-stratification techniques were implemented to ensure the representativeness of the data at the population level [1]. Post-stratification is a well-known sampling tool and is typically used with observational data from mobile phone data [2] or social media [3] to study transportation, mobility or segregation in cities. Specifically, we re-weight our results by census tract using the ratio of smartphone users to the true population in each area.

Let's denote w_α the ratio of the population of census tract α to the population detected in our data. For census block groups with fewer than 10 smartphone residents, the expansion factor w_α is set to 0 to ensure that we do not overweight users that are not representative for a given census block. Then we can weight our results by that expansion factor. For example, for average distance travelled in a city c by user the unweighted version is:

$$d^c = \frac{1}{M} \sum_{\alpha \in c} d_\alpha$$

where the sum runs over all census tracts α in city c and M is the number of users in our dataset, while the post-stratified one is

$$\hat{d}^c = \frac{1}{N} \sum_{\alpha \in c} w_\alpha d_\alpha$$

where N is the total population in the city. Similar expressions are used for the average number of bouts in each city.

Supplementary Note 2 Sensitivity analysis for detection of walks

To detect walk activity from the mobility data we used a methodology based on speed threshold and map-matching techniques [4, 5]. Walk bouts were extracted from consecutive locations in which the speed between them did not exceed typical walking speeds (2 meters/second) [6, 5]. Due to the geospatial accuracy of the data, we discarded walks of distance less than 50 meters. This also minimizes the potential

systematic error when calculating distances using GPS traces [7]. Since our walks are longer than 50 meters and assuming we have more than 3 locations in the path, using the results of [7] we estimate that systematic error in the distance to be always below 10

To minimize the impact of trajectories that could be misinterpreted as walking behavior, we only considered trajectories with geolocations that happened close to potential pedestrian areas. Using Open Street Maps we define those areas as those up to 20 meters from a secondary or tertiary road, residential or living streets, pedestrian, footway, track or path [8]. This definition excludes highways, motorways or trunks, where slow traffic or congestion could be mistaken as walks. We also considered those geolocations that happened within parks. Note that this strict definition of areas in which walks are possible exclude some of them where walking activity could be happening, like in parking areas, buildings or other large indoor spaces. However, our results do not depend on this restriction. As we can see in figure 4, the main results of our study considering all detected walks are very similar when we include only walks near a pedestrian areas.

We have also tested the sensitivity of our results towards the possibility that our detected walks are part of other mobility modalities. For example, a trajectory of more than 50 meters at a very slow speed could be part of a driving trajectory close to a stop, intersection or during a traffic jam. To this end, we have calculated the movements directly before and after (1 minute difference) our detected walks. Figure 5 show our results when we discard walks that happen just before, after or during faster movements (from 2 meters/sec to 10 meters/sec).

Finally, our results do not depend significantly on the 2 meters/sec threshold used to detect walks. As we can see in figures 6 (1.8 meters/sec) and 7 (2.2 meters/sec) our results are basically the same using different speed thresholds.

Supplementary Note 3 Comparison with other datasets

To validate our results externally, we have compared them with Apple mobility trends [9] regarding walking activity. Apple data does not reflect direct walking activity, but rather it represents the relative request volume of Apple Maps routes in the categories of driving, walking and public transportation. Thus, it might miss a large fraction of utilitarian walks for which users do not typically need routing instructions. Furthermore, the data does not contain demographic information and Apple Maps is only available on Apple devices so it is unknown whether their indices are representative for the entire population [10]. Despite those caveats, the comparison of our data with Apple mobility trends is very good (see Figure 8) for the 10 cities studied, particularly for the relative decay of walking activity directly after the lockdown and the level of recovery of walking activity after lockdown. We also note that there are some moderate deviations between the two datasets in the recovery element (e.g. Dallas or Los Angeles). This could be partly due to the well known problems that Apple mobility data has in places like California, where for example, driving data during COVID-19 is reported 25-50% in excess when comparing it with traffic data from the local government [10].

Supplementary Note 4 Spatial regression models

Because our variables of the multivariate regression model [see Equation (1) in the main text] are aggregated by census tracks as observational units, spatial dependence of observations may potentially bias the regression results. This is indeed the case and we observe a small spatial autocorrelation of the residuals for our linear regression models [see Equation (1) in the main text] before (Moran's I = 0.37) and after the COVID response (Moran's I = 0.21). To account for that spatial autocorrelation we modified our multivariate regression model to account for any potential spatial dependence of the walking behavior [11].

Specifically, the *Spatial Autoregressive Regression* (SAR) we use is

$$\bar{d}_{\alpha,i} \sim \sum_{\beta\in i} w_{\alpha\beta}\bar{d}_{\alpha,i} \tag{1}$$
$$+\text{income}_{\alpha,i} + \text{black}_{\alpha,i} + \text{public transportation}_{\alpha,i}$$
$$+\text{older } 64_{\alpha,i} + \text{park}_{\alpha,i} + \text{obesity}_{\alpha,i} + \text{MSA}_i + \varepsilon_{\alpha,i} \tag{2}$$

where α is the census tract in city i and $w_{\alpha\beta}$ is the spatial weighted matrix (we use first order queen contiguity). Table 2 shows the comparison between the Ordinary Least Square (OLS) and the SAR. Note that the OLS results slightly differ from the ones reported in the main text since for the comparison with the SAR model, we only consider census tracts which are neighbors with other areas. As expected, the spatial models yield to better models than OLS, but the gain is small both in R^2 and RSME. On the other hand the coefficients for the different demographic and environmental variables are consistent (same sign, statistical significance and order of magnitude). These comparison shows that, although our walking activity has a spatial correlation, the results presented in the paper do not depend significantly on that spatial structure. We have selected to report the OLS results in the main text because of that reason and to simplify our discussion.

Supplementary Note 5 List of software used

Analysis was conducted in R [12] using the following packages:

- Package `data.table` [13] for the load and transformation of the data tables.
- Package `ggplot2` [14] and `patchwork`[15] were used in the visualizations.
- Access to the Census API was done using the `tidycensus` [16] and `totalcensus` [17] packages. Boundaries of the Census Block Groups were obtained from the Census API using the `tigris` [18] package.
- Packages `sf` [19], `osrm` [20], and `osmdata`[21] were use to manipulate spatial data and the Open Street Map data.
- Spatial regression models were done using the `spatialreg`package [22]
- Regression tables were prepared using the `stargazer` package [23].

Supplementary References

[1] Salganik, M. J. *Bit by bit: Social research in the digital age* (Princeton University Press, 2019).

[2] Jiang, S. *et al.* The timegeo modeling framework for urban mobility without travel surveys. *Proceedings of the National Academy of Sciences* **113**, E5370–E5378 (2016).

[3] Wang, Q., Phillips, N. E., Small, M. L. & Sampson, R. J. Urban mobility and neighborhood isolation in America's 50 largest cities. *Proceedings of the National Academy of Sciences* **115**, 7735–7740 (2018).

[4] Nguyen, M. H., Armoogum, J., Madre, J.-L. & Garcia, C. Reviewing trip purpose imputation in GPS-based travel surveys. *Journal of Traffic and Transportation Engineering (English Edition)* **7**, 395–412 (2020).

[5] Stenneth, L., Wolfson, O., Yu, P. S. & Xu, B. Transportation mode detection using mobile phones and gis information. In *Proceedings of the 19th ACM SIGSPATIAL international conference on advances in geographic information systems*, 54–63 (2011).

[6] Cho, G.-H., Rodriguez, D. A. & Evenson, K. R. Identifying walking trips using gps data. *Medicine & Science in Sports & Exercise* **43**, 365–372 (2011).

[7] Ranacher, P., Brunauer, R., Trutschnig, W., Spek, S. V. d. & Reich, S. Why GPS makes distances bigger than they are. *International Journal of Geographical Information Science* **30**, 316–333 (2015).

[8] OpenStreetMap contributors. USA dump retrieved from https://planet.osm.org. `https://www.openstreetmap.org` (2020).

[9] Apple Inc. Apple mobility trends report. https://covid19.apple.com/mobility (2020). Retrieved: 2020-10-05.

[10] Gensheimer, J., Turner, A., Shekhar, A., Wenzel, A. & Chen, J. What are different measures of mobility changes telling us about emissions during the covid-19 pandemic? *Earth and Space Science Open Archive* 11 (2020). URL `https://doi.org/10.1002/essoar.10504783.1`.

[11] Anselin, L. & Bera, A. K. Introduction to spatial econometrics. *Handbook of applied economic statistics* **237** (1998).

[12] R Core Team. *R: A Language and Environment for Statistical Computing*. R Foundation for Statistical Computing, Vienna, Austria (2020). URL `https://www.R-project.org/`.

[13] Dowle, M. & Srinivasan, A. *data.table: Extension of 'data.frame'* (2020). URL `https://CRAN.R-project.org/package=data.table`. R package version 1.13.4.

[14] Wickham, H. *ggplot2: Elegant Graphics for Data Analysis* (Springer-Verlag New York, 2016). URL `https://ggplot2.tidyverse.org`.

[15] Pedersen, T. L. *patchwork: The Composer of Plots* (2020). URL `https://CRAN.R-project.org/package=patchwork`. R package version 1.1.0.

[16] Walker, K. & Herman, M. *tidycensus: Load US Census Boundary and Attribute Data as 'tidyverse' and 'sf'-Ready Data Frames* (2020). URL `https://CRAN.R-project.org/package=tidycensus`. R package version 0.10.2.

[17] Li, G. *totalcensus: Extract Decennial Census and American Community Survey Data* (2020). URL `https://CRAN.R-project.org/package=totalcensus`. R package version 0.6.3.

[18] Walker, K. *tigris: Load Census TIGER/Line Shapefiles* (2020). URL `https://CRAN.R-project.org/package=tigris`. R package version 1.0.

[19] Pebesma, E. Simple Features for R: Standardized Support for Spatial Vector Data. *The R Journal* **10**, 439–446 (2018). URL `https://doi.org/10.32614/RJ-2018-009`.

[20] Giraud, T. *osrm: Interface Between R and the OpenStreetMap-Based Routing Service OSRM* (2020). URL `https://CRAN.R-project.org/package=osrm`. R package version 3.4.0.

[21] Padgham, M., Rudis, B., Lovelace, R. & Salmon, M. osmdata. *The Journal of Open Source Software* **2** (2017). URL `https://doi.org/10.21105/joss.00305`.

[22] Bivand, R. & Piras, G. Comparing implementations of estimation methods for spatial econometrics. *Journal of Statistical Software, Articles* **63**, 1–36 (2015). URL `https://www.jstatsoft.org/v063/i18`.

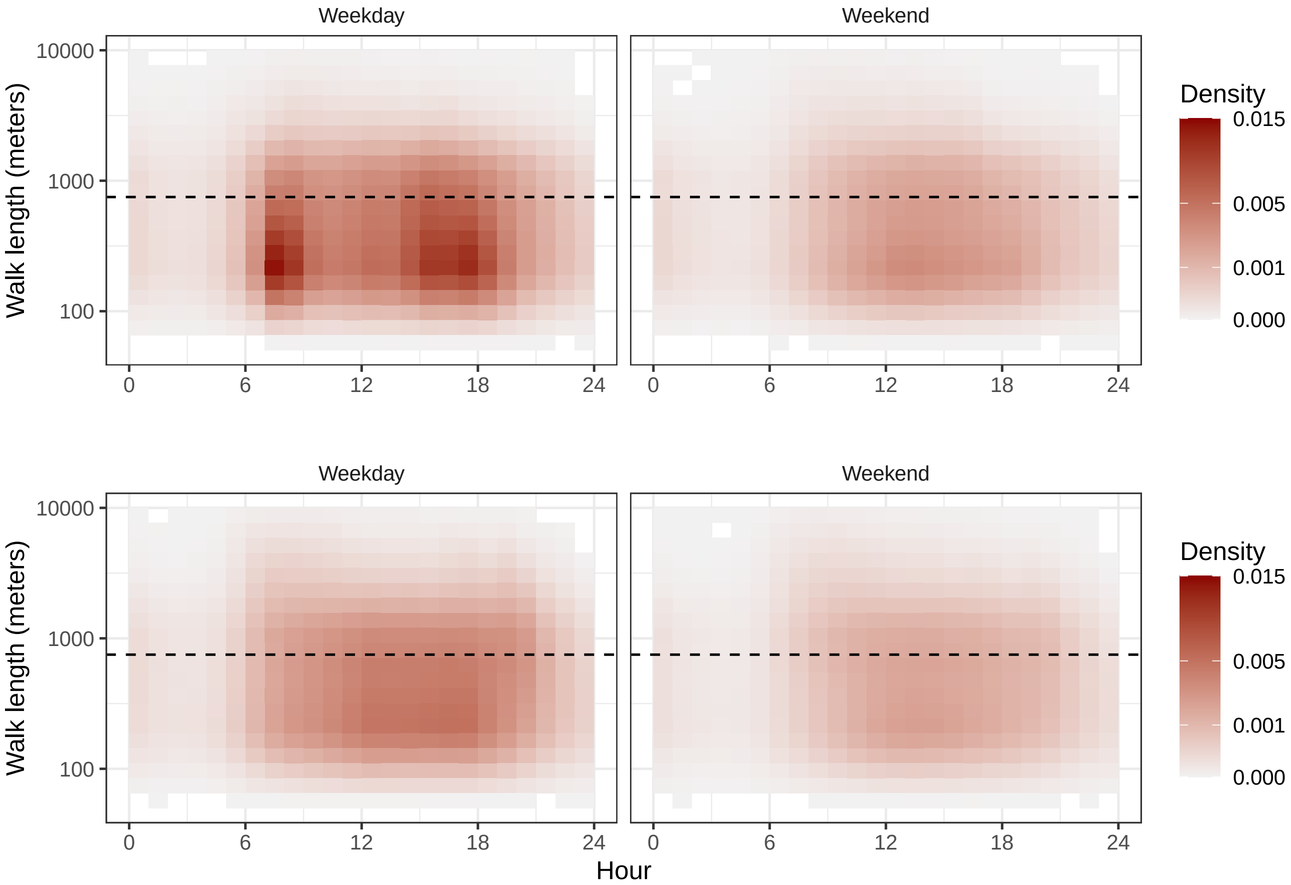

Supplementary Figure 1: Panels A) to D) show the distribution of walks by hour of the day and length (in meters) for the walks before (upper panels) and after (lower panels) COVID-19 measures and for weekdays and weekends. Dashed line correspond to 750 meters.

[23] Hlavac, M. *stargazer: Well-Formatted Regression and Summary Statistics Tables*. Central European Labour Studies Institute (CELSI), Bratislava, Slovakia (2018). URL `https://CRAN.R-project.org/package=stargazer`. R package version 5.2.2.

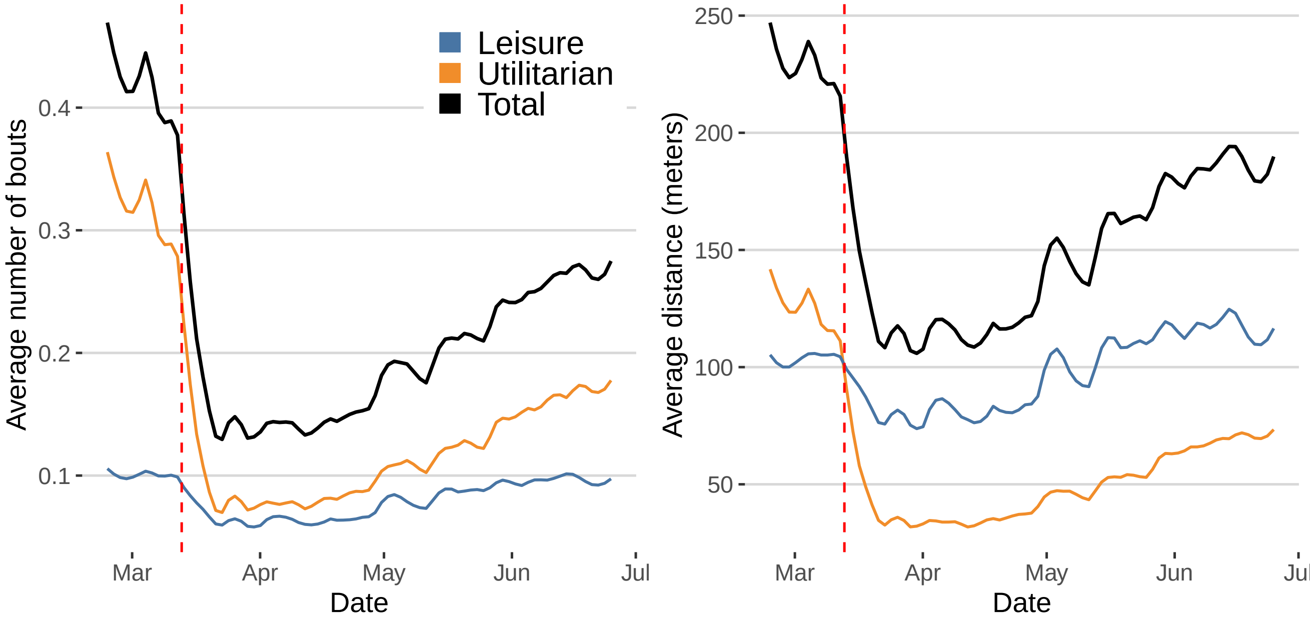

Supplementary Figure 2: Time series of the average number of walks (left) and distance (right) by user and day using 500 meters as the threshold for utilitarian walks.

Supplementary Table 1: Percentage and properties of walks by destination. Ordered by average distance.

Destination	Percentage of walks	Average Distance (meters)	Average Duration (minutes)
No destination	61.96	565.14	9.12
Coffee / Tea	0.69	529.94	8.30
Work	2.94	523.86	8.19
City / Outdoors	3.09	522.27	8.16
Arts / Museum	1.13	519.09	8.16
Sports	0.29	515.14	8.33
Entertainment	2.10	511.38	8.03
Food	7.93	509.97	8.00
College	1.20	507.20	8.02
Grocery	1.12	499.98	7.93
Transportation	5.19	499.97	7.70
Service	6.42	496.10	7.82
Shopping	2.76	489.53	7.77
Health	1.97	488.16	7.73
School	0.70	462.91	7.31

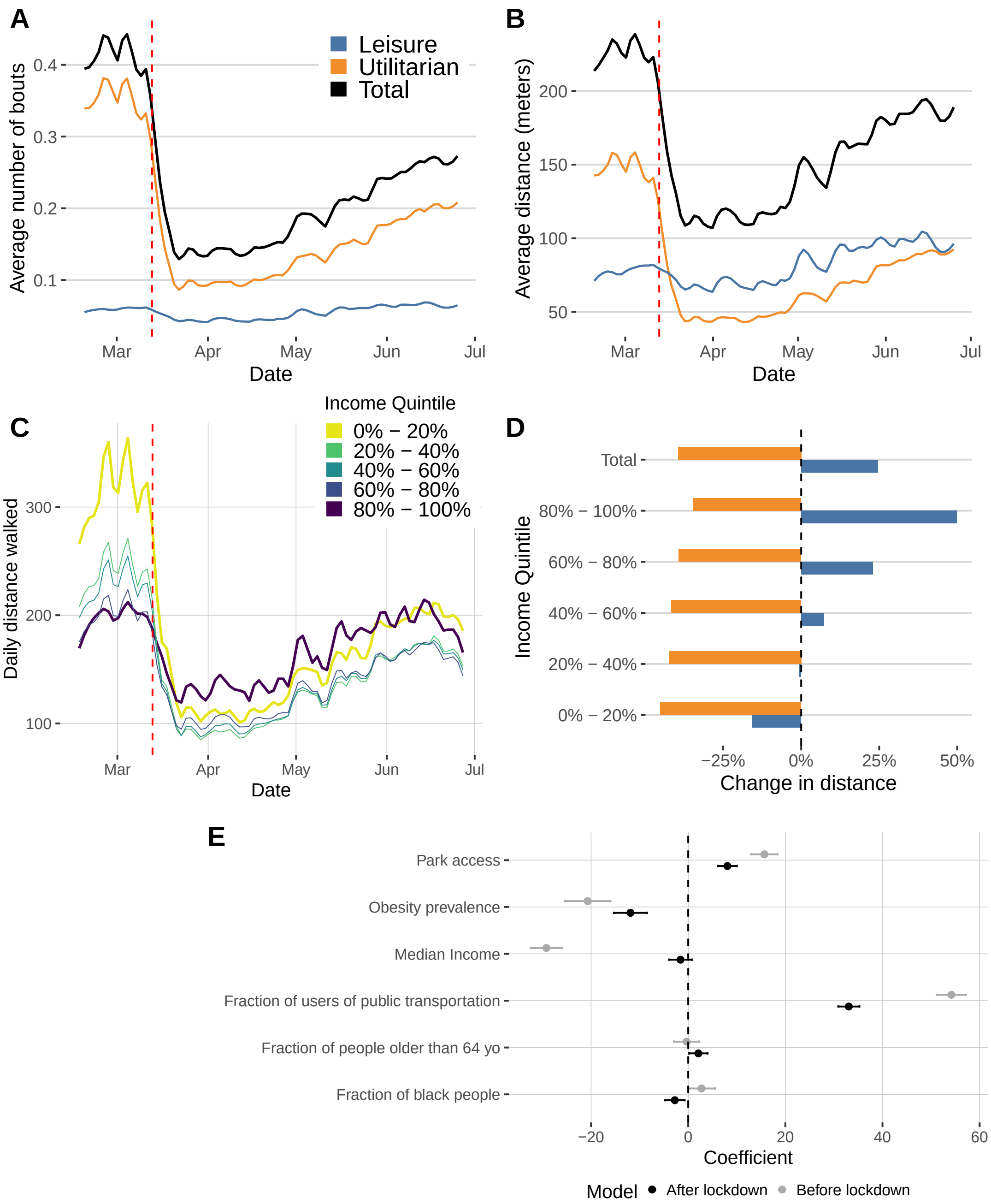

Supplementary Figure 3: Summary of the results in the main paper (for comparison with sensitivity analysis). A) Total (black) daily average number of bouts of walking by user in the 10 metropolitan areas, compared with those for utilitarian and leisure walks. B) Same as in A) but for total distance walked. C) Average daily distance walked by different income sub-groups (quintiles) D) Change in utilitarian or leisure walking for the same income sub-groups, including the total. E) Coefficient results for the multivariate model (see Table 1 in the main text) to explain the average distance walked (in meters) by individuals in different areas as a function of the demographic and environment properties of that area. Bars correspond to their confidence interval given by the standard error of the coefficients. The multivariate model was obtained for $N = 9,111$ different census tracts.

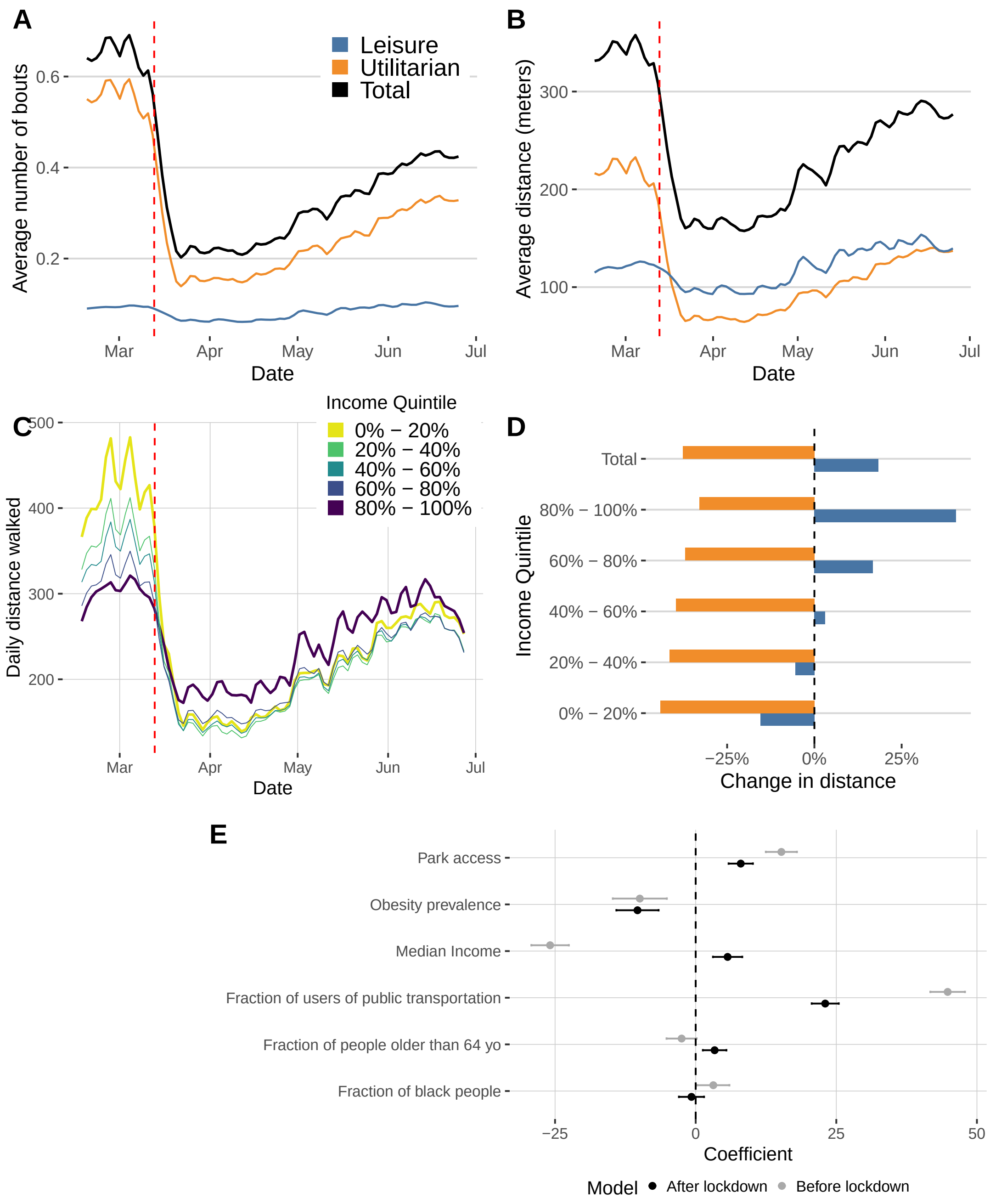

Supplementary Figure 4: Same as in figure 3 but considering all walks independently of where they happen. See Figure 3 caption for other details.

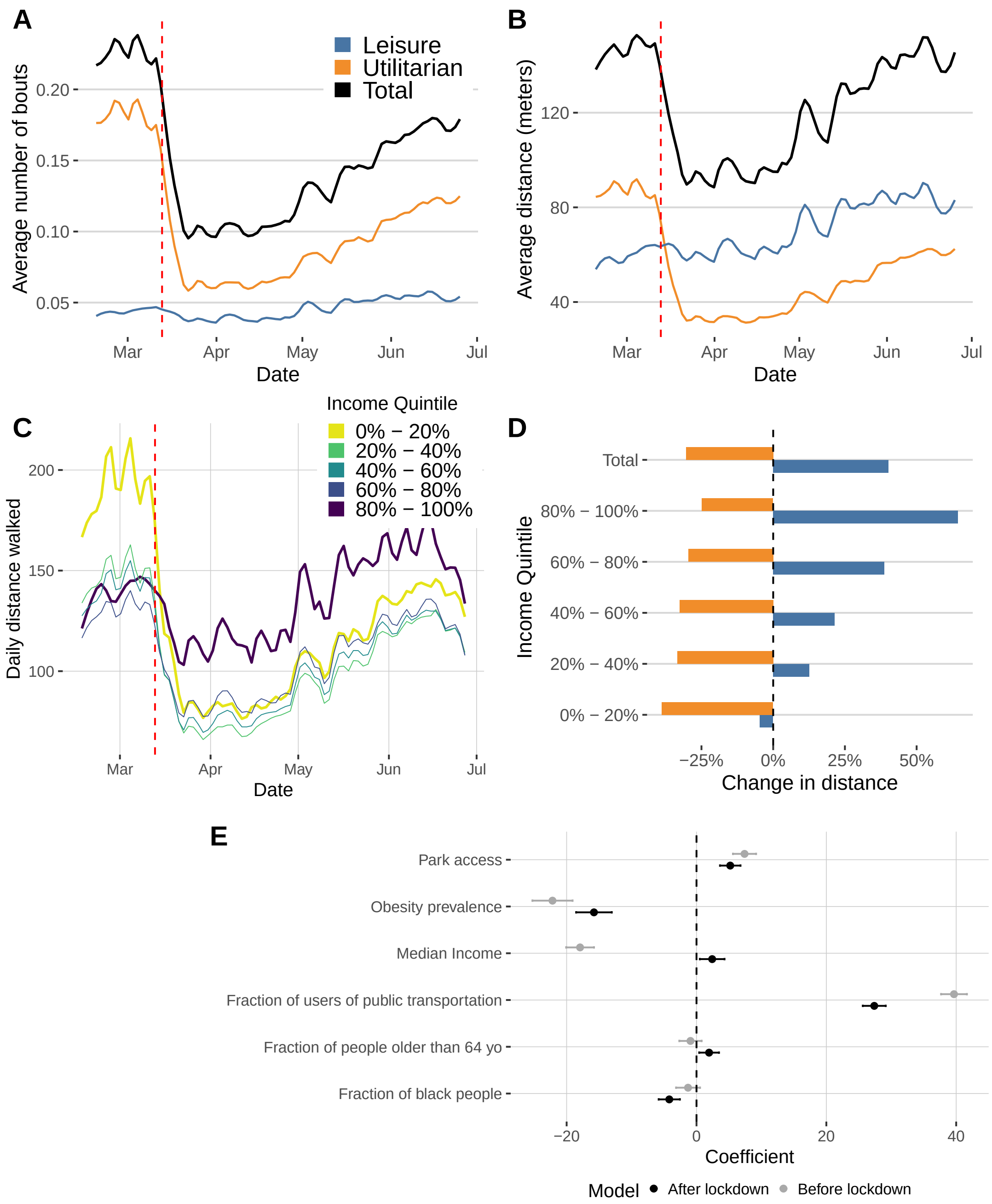

Supplementary Figure 5: Same as in figure 3 but discarding walks that happen just before, after or during detected movements of larger speed. See Figure 3 caption for other details.

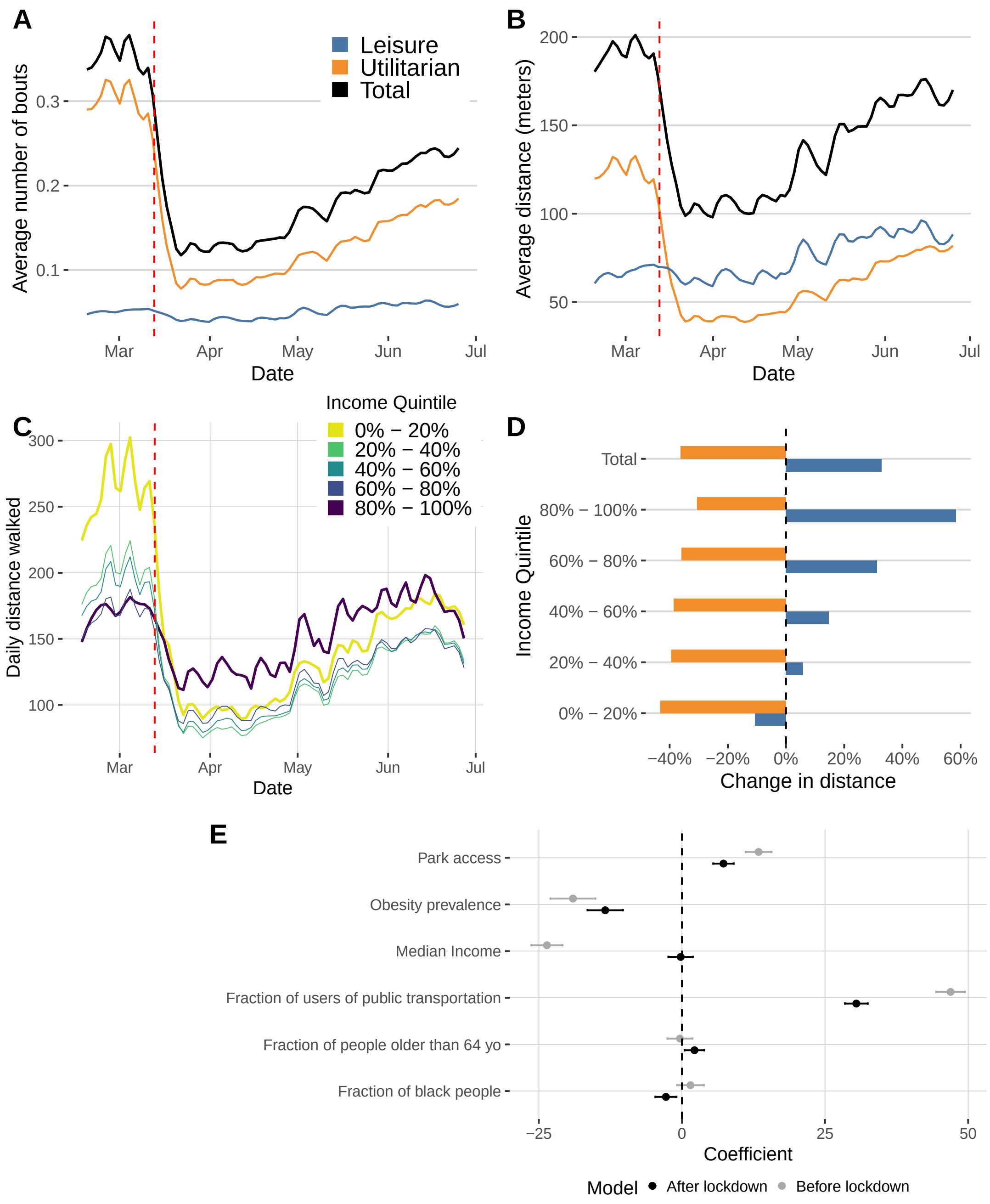

Supplementary Figure 6: Same as in figure 3 using 1.8 meters/second as the threshold for speed between consecutive points. See Figure 3 caption for other details.

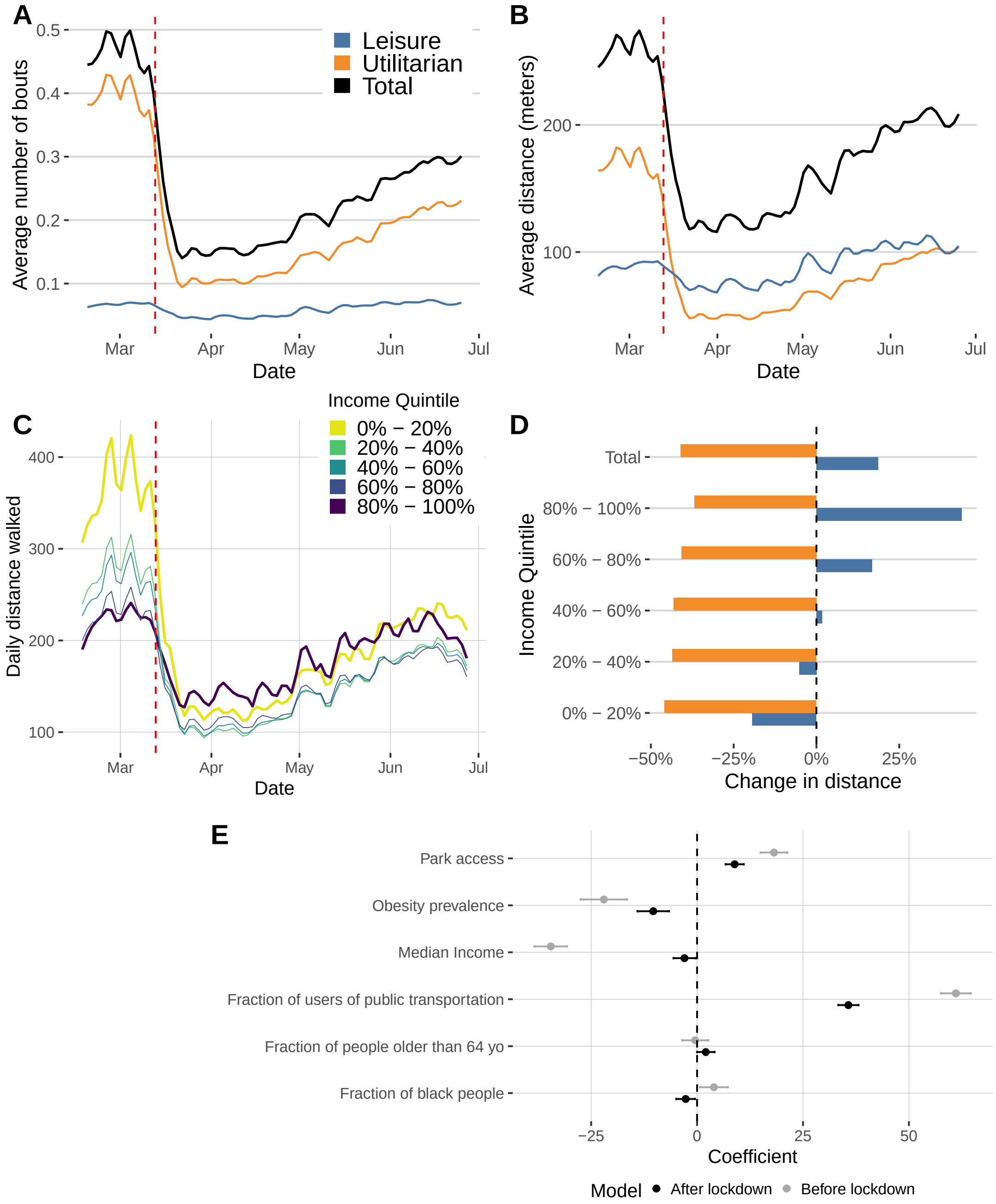

Supplementary Figure 7: Same as in figure 3 using 2.2 meters/second as the threshold for speed between consecutive points. See Figure 3 caption for other details.

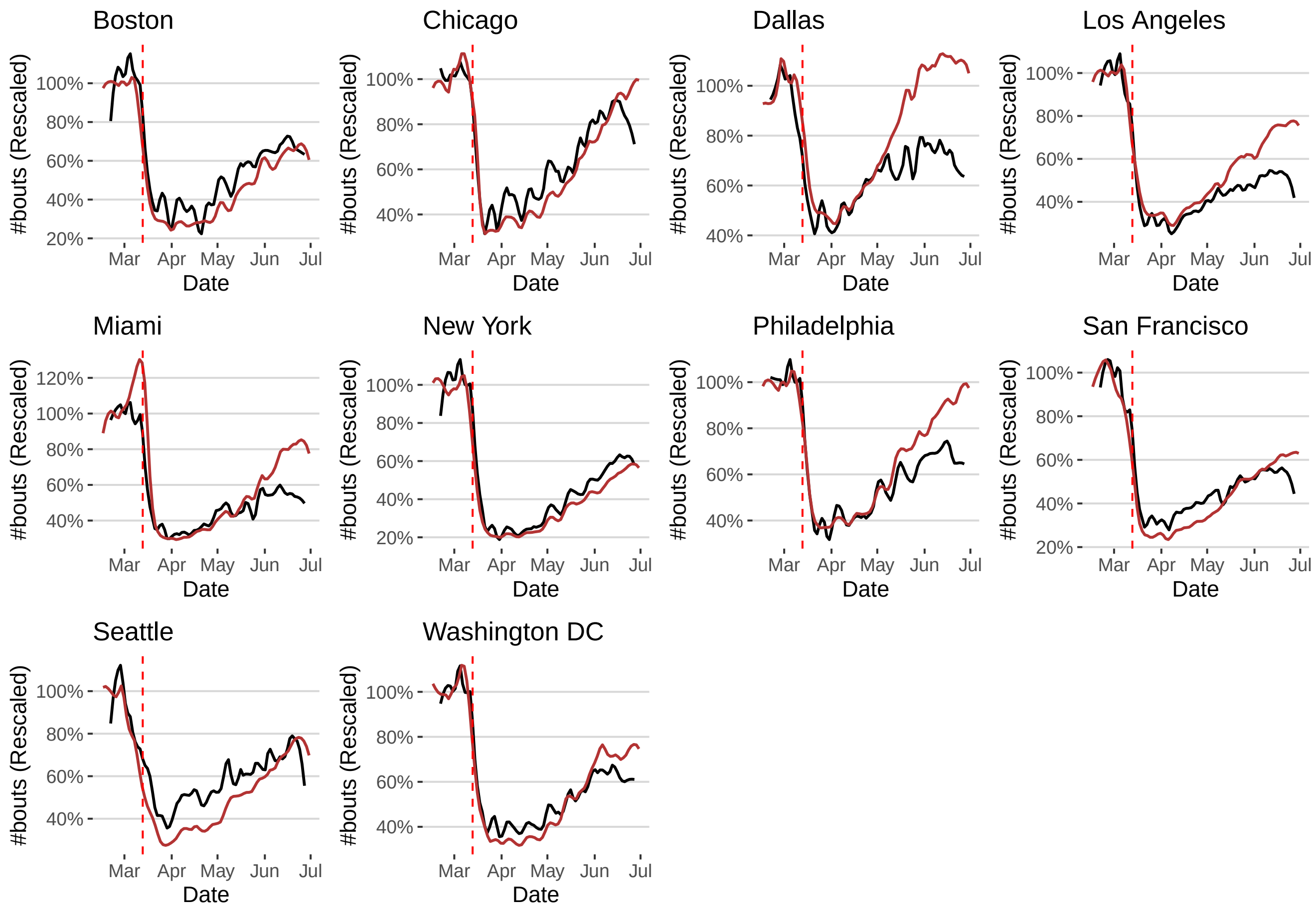

Supplementary Figure 8: Comparison between the number of walking bouts detected in our data set (black) and Apple mobility trends index (red). Both are re-scaled to levels before March 1st 2020.

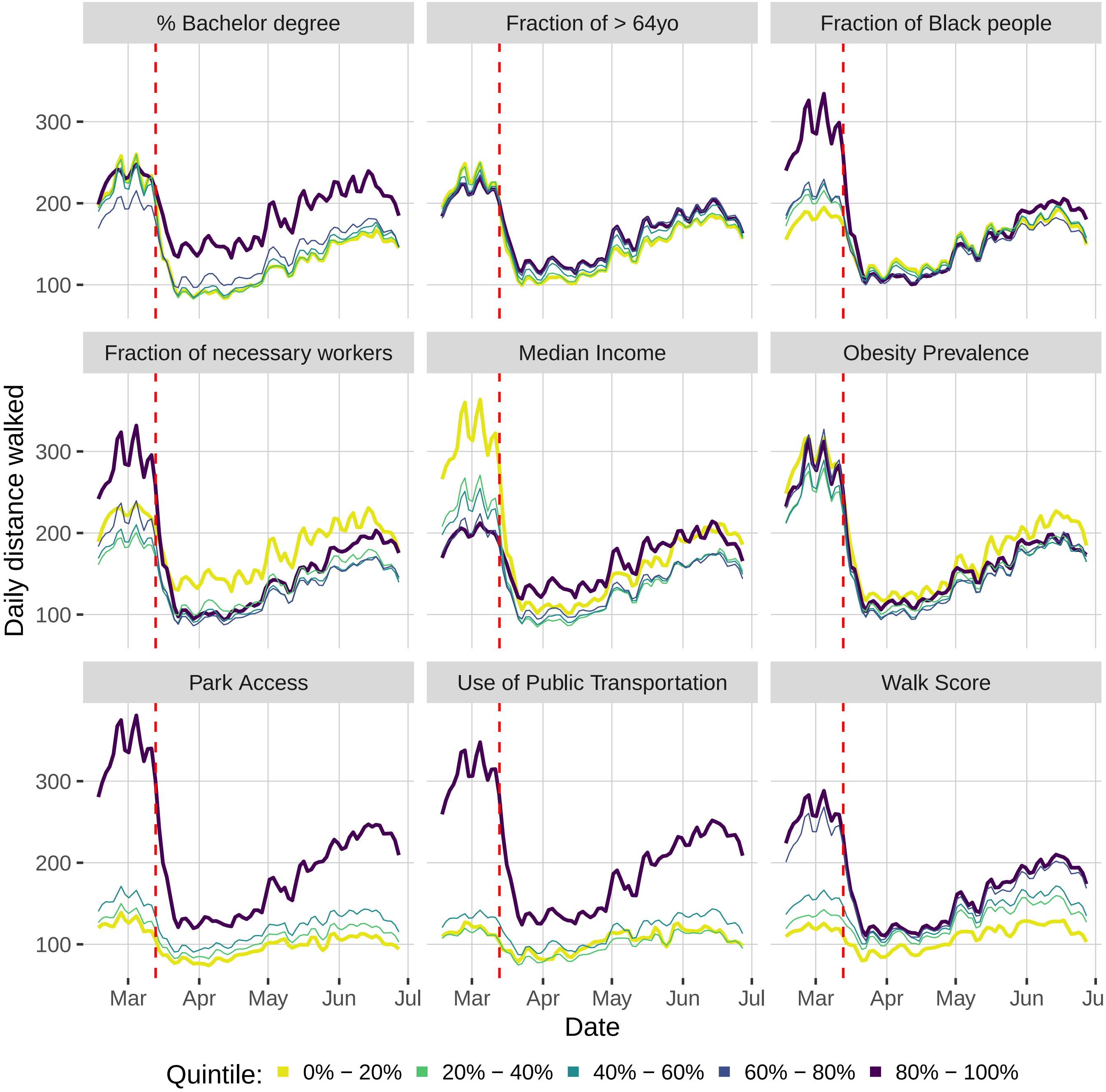

Supplementary Figure 9: Evolution of the average distance walked by day by different sub-groups (quintiles) for different demographic (education, fraction of more than 64yo people, fraction of Black people, fraction of necessary workers, median income), environment (park access, walk score) and health (obesity prevalence) traits.

Supplementary Table 2: Comparison between the Ordinary Least Squares (OLS) and Spatial Auto-regressive Regression (SAR) multivariable models to explain the average distance walked by individuals in different areas as a function of the demographics properties of that area. ρ is the coefficient for the spatial auto-regressive part. To compare OLS and SAR we show the R^2 (OLS) and pseudo-R^2 for SAR and the RMSE for both of them.

	Average distance walked:			
	Before COVID response		After COVID response	
	OLS	*SAR*	*OLS*	*SAR*
ρ (spatial autorregressive coeff)		0.618^{***}		0.434^{***}
		(0.010)		(0.013)
Median income	-30.091^{***}	-12.566^{***}	-1.750	0.823
	(1.678)	(1.384)	(1.148)	(1.062)
Fraction of Black people	3.344^{**}	1.548	-2.793^{**}	-2.271^{**}
	(1.599)	(1.304)	(1.094)	(1.012)
Fraction of users of public transp.	64.583^{***}	24.061^{***}	37.292^{***}	21.952^{***}
	(1.815)	(1.567)	(1.242)	(1.210)
Fraction of > 64yo	-0.446	-1.491	1.716^{**}	1.127
	(1.179)	(0.962)	(0.807)	(0.746)
Park access	15.034^{***}	6.990^{***}	7.266^{***}	4.452^{***}
	(1.315)	(1.076)	(0.900)	(0.834)
Obesity prevalence	-21.382^{***}	-7.520^{***}	-11.634^{***}	-4.354^{***}
	(2.374)	(1.940)	(1.625)	(1.509)
Constant	346.708^{***}	130.865^{***}	238.404^{***}	133.342^{***}
	(5.581)	(5.794)	(3.819)	(4.703)
City fixed factor	YES	YES	YES	YES
Observations	8,999	8,999	8,999	8,999
R^2	0.762	$0.842^{\dagger}$	0.696	$0.740^{\dagger}$
RMSE	100.6	82.08	68.83	63.69
Note:	† Pseudo-R^2, *p<0.1; **p<0.05; ***p<0.01			
